# Echoes of the Month

**Published:** 1923-11

**Authors:** 


### ECHOES OF THE MONTH.

### DRUG ADDICTION IN ENGLAND.

In his Norman Kerr Memorial Lecture on "Drug Addiction" Sir William Willcox said that the commonest cause of addiction in the citizen classes was undoubtedly the prescription of the drug for medical purposes. It was to be feared that after operations drugs were too often given as a routine practice, without sufficient regard to the dangers of toxic action and the development of addiction disease. The continued use of drugs for insomnia was a method of treatment harmful for the cure of the condition, and patients should be led to take that view. The terrors of insomnia were incomparable to those of drug addiction, and a little sleep following natural methods of treatment was of far greater benefit than the more prolonged abnormal sleep produced by narcotic drugs. As to the general lines of treatment the only hopeful method was one which recognised that drug addiction was a disease requiring the same careful attention to detail that any other disease would receive. The general standard of health should be raised to as high a pitch as possible, and any predisposing factors should be dealt with. In that way the prospect of cure was as hopeful as in the case of other diseases. Where the general health was good and the period of addiction short, sudden withdrawal of the drug was advisable. He believed the dangers of sudden withdrawal had been exaggerated.

### DANGER OF BAD TEETH.

In a lecture delivered at the Dental Exhibition, Dr. Butterfield said that pre-natal dental care ought to be compulsory for all expectant mothers, but the difficulty of securing compulsory vaccination showed that this was not practicable. Nor was it possible, unfortunately, to make dental treatment compulsory in the schools. How many cases were known of unhealthy tonsils co-existing with healthy teeth? He quoted the case of an athlete that had baffled the doctors for ten years. Every now and again he would feel a pain in the shoulder, and three days later a white spot would appear on the first finger. Eventually he visited a dentist, who found that one of the teeth had a cavity infected with pyorrhœa, and when this was cleaned up there was no recurrence of the trouble in the finger. Often the first symptom a patient had was a pain in the knee joint, owing to the *streptococcus salivaris* having travelled through the system to that joint.

### BOARDING OUT MENTAL DEFECTIVES.

An experiment is being tried by the committee responsible for carrying out the Mental Deficiency Act in Birmingham by boarding out, under approved guardians, a number of feeble-minded young men and women in the country around Birmingham. There is a scarcity of institutional accommodation, and the hope is entertained that many of these feeble-minded persons, who are described as "high grade" of their class, and "near the line of mental normality," may benefit considerably. The youths are, it is stated, quiet and easily controlled, and are capable of performing efficiently simple duties on the land; while the young women whom it is desired to board out are able to do useful domestic work, and to give effective help on poultry farms and in the lighter branches of agriculture. The employer receiving a feeble-minded person of this grade is required to act "in loco parentis" and give an undertaking not to allow him or her "to go out alone." The authorities pay a sum varying from 10s. to 15s. per week to the employer, who has, of course, the benefit of the work of the feeble-minded person.

### A RESERVOIR OF DISEASE.

The health situation around the Pacific was discussed at the Pan-Pacific Science Congress in Australia last month. The Pacific is without contradiction a reservoir of disease. There is practically no disease that is not at work in some part of the huge ocean at any given date. Cholera, plague (which in New Caledonia is ineradicable), malaria, leprosy, beri-beri (sleeping sickness), influenza (which is now one of the worst death-recorders, are all well known. What, however, the foreign health authorities seem to be afraid of is yellow fever, although since the Spanish left the Philippines there has been no epidemic. But the yellow fever mosquito, the stegomya, is common practically all over the Pacific, and if the disease did get entry again it might well be a very difficult task to gain the upper hand over it. Actually, the Australian authorities are not perturbed. They consider, and in view of the statistics probably rightly, that yellow fever is so unlikely that it is not yet worth bothering about, whilst influenza is taking a terrific toll of life, and there appears to be no remedy, nor even a preventive. The losses among the native races in the islands during the epidemic of 1920 and 1921 were enormous. Among the whites, both in New Zealand and in Australia, the death-rate more than doubled the normal.

### FRENCH SURGEONS AND THE VORONOFF METHOD.

At the recent Congress of Surgery held in Paris, eminent surgeons upheld Dr. Voronoff's method of rejuvenating human beings by grafting the interstitial glands of chimpanzees upon them. Dr. Dartigues, of the Paris Faculty of Medicine, gave a lecture on the technique of the grafting operation, illustrated by photographs. He mentioned also the need for the protection of chimpanzees against hunters who destroyed them simply for sport, since they were growing extremely scarce just at a time when humanity had discovered its greatest need of them. M. Baudet described the results which he had obtained in grafting operations performed on a business man, a professor, a writer, and an engineer. Before treatment the business man had found himself no longer able to exercise efficient supervision over his affairs, the professor was losing mental grip to such an extent that he had tendered his resignation, and the engineer was unable to apply himself to serious work for more than half-an-hour at a time. In every case the Voronoff treatment had given them a renewal of vigorous youth from the mental point of view.

### VILLAGE FREED FROM GOITRE.

The introduction of iodine into children's diet as an effective means of combating goitre is the subject of an experiment now being conducted in the kitchens of the American relief administration in Austria. The best method employed, according to a recent issue of the Administration's Bulletin, has been the substitution of iodine salt for table salt. This method was previously used with success in Switzerland, where goitre is most prevalent, by Professor Wagner-Jauregg. Experiments in the schools were supplemented by systematic internal use of iodine. The Swiss physician, Dr. Bayard, on the other hand, instead of ordinary cooking salt, gave a salt supplemented with iodine to the population of an entire village for a period of six months, with the consequence that the village was practically freed from goitre.

### A CURIOUS CURE FOR RINGWORM.

In a recent lecture at Nottingham University College Dr. E. F. Skinner, of Sheffield Children's Hospital, gave an instance of curious remedies for diseases. Many of the skin diseases from which Sheffield children suffered, he said, were attributed by mothers to fish and chips, cheese and beer. A curious cure for ringworm which he had encountered was a mixture of ink and tobacco, and it was remarkable that this had effected cures. This was due to the fact that ringworm was caused by a fungoid growth like mushrooms, and the only way to destroy it was by boiling. Obviously, it was impossible to boil a baby, so the skin had to be artificially inflamed. This was what mixture of ink and tobacco accomplished.

### FINSEN'S OWN CITY.

The Medical Correspondent of the *Observer* has been visiting Copenhagen in order to see at first hand something of the Finsen treatment. "It is good," he writes, "to see the great work of a great man rightly carried on after his death. Not merely do we find an allegorical group of human figures stretching to the light as the Finsen Memorial on one of the boulevards of this city, but also the magnificent Finsen Medical Light Institute, which comprises a large and perfectly equipped hospital for in-patients suffering not only from skin tuberculosis, but also from all the forms we still call 'surgical'; a busy out-patient department; and an important laboratory, where experts are engaged in studying the *modus operandi* of the light. Hither repair practically all cases of skin and 'surgical' tuberculosis in Denmark and large numbers from other countries, including our own. It is difficult to say whether one admires the clinical or the research work the more. In the long run, doubtless, the latter is the more important. It suggests, at the present stage, that we shall do well not to pin our faith to any one kind of artificial lamp."

### HOW TO GET CLEAN MILK.

Lecturing on "Our Milk Supply" at Birmingham Midland Institute, Dr. Davidson, Assistant M.O.H. for the city, said that if the purchaser would only insist on having the article up to standard, and deal only with those tradesmen who could supply it, he would have done more towards getting a good milk supply in a short time than could be done by any other method. There was great activity in Birmingham, where some firms were spending large sums of money in remodelling their premises and studying the problem in all its aspects so as to produce high quality milk. One Birmingham firm paid a bonus of a penny per gallon for the cleanest milk delivered to them each month. The three principal factors which spoilt milk were external contamination, high temperature, and age, and to remove these they must concentrate on clean production, clean methods, cooling and speed in delivery. Some of the evidence as to the prevalence of tuberculosis in milk was rather startling. During the past 15 years 65 new herds—representing about 1,500 animals—were tested under the Birmingham Corporation scheme for assisting the eradication of tuberculosis from herds that supplied milk to the city. Thirty-seven per cent. were found to be affected in some degree; in all probability the percentage of affected animals throughout the country was higher. It was generally believed that bovine tuberculosis was the source of the majority of cases of bone, joint and gland tuberculosis in children.

### TESTS FOR BABIES.

Psychological tests for babies are the fashion in New York, and are showing that many infants are far in advance of their years. Experts have agreed so far to the following determinants of normality:—At six months a normal child should be able to listen to a watch ticking. At seven months the same child should be able to take a few swallows from a cup of liquid held to its lips. At nine months the child should be able to pick up a piece of sugar or a crumb, using the thumb and forefinger. At twelve months a bell should be tapped, and upon the bell ringing the normal child will repeat the tapping to hear the ring again. At fifteen months a normal child, given a corked bottle, ought to be able to pull the cork out, provided the cork is not so tightly inserted as to compel the use of a corkscrew.

### ADENOIDS IN BRITISH COMMUNITIES.

The *Medical Press* is asking why adenoids are a typical ailment of the British Communities. The disease is as common in villages as in towns and would appear to be due to some error in diet: "In whatever region of the world British communities are met with, whether it be in Australasia, Canada, or South Africa, there British children develop luxuriant adenoids. One of the worst cases of adenoids I have come across was in a child who had just returned from South Africa. He had been brought up on the veldt, and it is notorious that, though rickets is rare in sunny Australia, adenoid disease is rampant among the white children. It is unknown among the unspoilt aborigines. Now what is the one environmental factor competent to produce so profound a structural change, which distinguishes these British communities from all others? I can only think of the dietetic factor; the chief cause of adenoid disease and its evil consequences is the flooding of the young stomach with starch in a form which does not compel mastication."

### "HEALTH HINTS FOR VACATIONISTS."

American holiday-makers who carried their radio receiving sets with them this summer are not suffering from lack of advice on health. Thirty broadcasting stations, in addition to the Naval Radio Station, Arlington, have been sending out "Health Hints for Vacationists," furnished by the Bureau of the United States Public Health Service at Washington. The most recent of these broadcasts on the subject of poison ivy was especially timely, since every summer brings its toll of suffering from this cause. The poison ivy is perhaps the plant most frequently encountered by the unsuspecting city visitor to the country, says the Public Health Service. The part of these plants to be feared is the resinous sap. When a plant is injured this sticky sap exudes. It comes in contact with the skin and sets up an irritation which is distinguished by its acute character. This irritation frequently begins between the fingers. A mild attack may be ushered in by a burning or itching of the skin. Within twenty-four hours after the skin is exposed to the poison of the plant, a red rash appears. This is followed by more or less swelling and itching, then small blisters filled with serum make their appearance. The parts of the body affected may swell to enormous proportions. The treatment of ivy poisoning is simple and easily administered. One of the best treatments is bathing with salt water. Sea water is best if it is available.

### A MUNICIPAL HOME FOR MOTHERS.

Bermondsey Borough Council has opened a convalescent home at Fairby Grange, Kent, in association with its maternity and child welfare work. It is a charming country house and will provide accommodation for the treatment of approximately 350 to 400 mothers annually. The standard charge will be 10s. a week, but in special cases admission may be free. It cannot, of course, be assumed that all those who need treatment will be able to avail themselves of the opportunity offered at Fairby Grange. Probably those whose need is greatest will be the least able to go away, either on account of the expense or because of the impossibility of leaving other young children at home. To meet this obstacle it has been decided to assess the charges to be made according to the circumstances of the family and to allow a mother to take one toddler in addition to her infant. The health visitors have been instructed to make the home known to all their mothers and to give every assistance to the applicants. The success of the scheme will be, to a large extent, dependent on their efforts.

### TREATMENT FOR SLEEPING SICKNESS.

Professor Klein and Dr. Fischer, two German scientists who have been engaged for the last year or two in applying the "Bayer 5" treatment for sleeping sickness in Northern Rhodesia and the Congo, have made a large number of cures. The treatment, said Professor Klein, consisted of from three to five injections, which were given as the condition of the patient warranted. The patient usually felt better after the first or second injection. The natives appeared to have great confidence in the Bayer treatment, and freely availed themselves of it. The cures which had been made among their fellow-men convinced them of its effectiveness.

### BLIND WORKERS FOR THE BLIND.

In its Annual Report the National Institute of the Blind records that last year a sum of £68,684 was expended in salaries, wages, etc., to 332 blind employees actively engaged in all branches of the Institute's work. The majority of the collecting staff are blind; embossed books are stereotyped by the blind; proofs are corrected by them; music is prepared by trained blind workers; practically the entire work of the Home Teaching branch is carried on by blind home teachers; blind telephonists and shorthand-typists are employed at Headquarters; several of the most important executive positions, such as the organisers of the Greater London Fund, the organist and director of music, the superintendent of the After-Care Department, the principal of the Massage School, are filled by blind men. The principle of employing the blind to carry on ameliorative work in connection with their fellows has been characterised by marked success, and largely assists also in solving the very grave question of unemployment.

### A "CHILDREN'S HOUSE" AT BOW.

A house called "The Children's House" has been opened at Bow, which is a combination of c èche and kindergarten, where babies can be left in safety during the mothers' working hours. The charges are necessarily very modest. For fourpence a day working mothers can secure for each child complete attention and suitable food for a whole day, and it is intended in the summer time to keep the children on the flat roof which has been laid out as a garden so that they may have their meals, their play and their sleep among the flowers. The most remarkable feature of the Children's House is undoubtedly the decoration. which has been designed and executed as a labour of love by some of the best known artists and sculptors in London.

